# “It’s not about wanting to be thin or look small, it’s about the way it feels”: an IPA analysis of social and sensory differences in autistic and non-autistic individuals with anorexia and their parents

**DOI:** 10.1186/s40337-023-00813-z

**Published:** 2023-06-05

**Authors:** Emy Nimbley, Karri Gillespie-Smith, Fiona Duffy, Ellen Maloney, Carrie Ballantyne, Helen Sharpe

**Affiliations:** 1 Department of Clinical Psychology in the School of Health in Social Science, University of Edinburgh, Old Medical School, Elsie Inglis Quadrangle, Teviot Place, Edinburgh, EH8 9AG Scotland, UK; 2 NHS Lothian Child and Adolescent Mental Health Services, Royal Edinburgh Hospital, Edinburgh, Scotland, UK; 3 School of Philosophy, Psychology and Language, University of Edinburgh, Edinburgh, Scotland, UK; 4 School of Education and Social Sciences, University of the West of Scotland, Glasgow, UK

**Keywords:** Autism, anorexia nervosa, Parents, Social, Sensory, IPA

## Abstract

**Background:**

Despite increasing evidence to support an overlap between autism and anorexia nervosa (AN), underlying mechanisms remain poorly understood. Social and sensory factors have emerged as promising targets in both autism and AN, however there remains scope to compare these differences across autistic and non-autistic experiences of AN. Drawing on dyadic multi-perspectives, this study explored experiences of social and sensory differences in autistic and non-autistic adults and their parents and/or carers.

**Methods:**

Using interpretative phenomenological analysis (IPA), dyadic interviews were conducted with 14 dyads, with seven autistic dyads and seven non-autistic dyads. Data analysis was subjected to a triangulation of interpretations: (1) the participants themselves; (2) a neurotypical researcher; (3) and an Autistic researcher with lived/living experience of AN.

**Results:**

IPA identified three themes in each group, with similarities and differences between autistic and non-autistic dyads. Similar themes were identified regarding the importance of social connectedness and socio-emotional difficulties, as well a common lack of trust in the social and sensory self and body. Autism-specific themes centred on feelings of social ‘defectiveness’, disparities between sensing and expressing certain cues, and lifelong, multi-sensory processing differences. Non-autistic themes reflected social comparisons and inadequacy, and sensitivities to the learning of ideals and behaviour through early experiences.

**Conclusions:**

While similarities were observed across both groups, there appeared to be notable differences in the perceived role and influence of social and sensory differences. These findings may have important implications on the delivery and modification of eating disorder interventions. Specifically, they suggest that while treatment targets may look similar, subtle differences in underlying mechanisms and approaches may be required for Autistic individuals with AN across sensory, emotion and communication-based interventions.

**Supplementary Information:**

The online version contains supplementary material available at 10.1186/s40337-023-00813-z.

## Introduction

Autism spectrum disorder (ASD, here on referred to as autism [1, 2]) is a neurodevelopmental condition, characterised by differences in social communication and interaction, sensory processing and rigid behaviours [3]. Anorexia nervosa (AN) is a severe eating disorder (ED), characterised by an intense fear of gaining weight, body shape disturbances and persistent attempts to restrict dietary intake [3]. Increasing research evidence suggests a significant overlap between autism and AN, with prevalence estimates range between 20 and 40% [4-6]. Furthermore, despite early concerns that the cognitive impact of starvation can mimic certain Autistic traits (e.g., [7]), several lines of evidence support a true etiological relationship; for example, Autistic traits have been found in those who have recovered from AN [8, 9] and are longitudinally associated with disordered eating in adolescence [10].

Autistic individuals with AN report more severe and enduring ED symptoms [11], poorer psychosocial outcomes [12, 13] and poorer treatment outcomes [8, 14]. They also report frustration with ED services [15] and a disparity between ED treatments and their individual needs [16, 17]. Despite this, Autistic individuals have been reported to conceptualise recovery in notably similar ways to non-autistic individuals [18]. Thus, to be able to further understand the co-occurrence of autism and AN and effectively support autistic individuals, a more comprehensive understanding of their similarities and differences in underlying mechanisms must be achieved. Several promising targets have been identified, including socio-emotional domains of emotion recognition [19] and theory of mind [20, 21]. Broader social difficulties, such as friendship difficulties, have also been implicated in autism [22] and AN [23], with a recent comparative study suggesting that reciprocal social interaction may help discriminate between autistic and non-autistic individuals and AN [24]. A recent qualitative model exploring restrictive EDs in Autistic women highlighted a broad range of possible autism-specific mechanisms, including differences in emotion perception, sensory processing, social interactions and relationships [25].

Both social and sensory factors therefore offer promising targets for untangling common and autism-specific mechanisms in the development of AN. Qualitative research allows for the generation of understanding rooted in the lived/living experience of the individual. These understandings can also be further informed by including multi-perspectives, such as those of parents and/or carers. Carers have expressed frustrations with ED services in similar ways as their children [26] and their perspectives have made an important contribution to our understanding thus far [16, 25]. However, this has typically taken the form of collecting perspectives individually, before drawing across interviews and considering shared and distinctive features. Dyadic approaches not only provide multiple perspectives but also allows for a real-time interaction of perspectives, creating novel opportunities to discuss ideas that may not have occurred to each individual separately [27, 28]. This study will therefore seek to explore social and sensory differences across autistic and non-autistic adults with current or lifetime history AN and their parents and/or carers. The study will seek to address the following research questions:

1. What social, sensory and other factors do autistic and non-autistic dyads perceive to play a role in their AN?
2. What are the similarities and differences in the factors identified by autistic and non-autistic dyads?

## Methods

### Participants

Dyads were recruited from the community through relevant ED and autism charities and organisations, and advertisements on social media platforms. Study adverts were also disseminated through internal mailing lists and waiting rooms within the University of Edinburgh. The inclusion criteria for participation were that both members of the dyad were fluent in English and over 18 years old. Autistic and non-autistic adults with current or lifetime history of AN were required to be UK-based and to have received a clinical diagnosis of AN, including atypical anorexia. Their parents or caregivers were required to be their mother, father or someone that taken on a caregiving role across development. Due to the broad focus of the study in exploring the role of social and sensory factors across AN trajectories, participants were not grouped into current or recovered, however this information was collected (Table 1). In total, 14 dyads were recruited. Seven dyads were recruited across each group (n = 28), reflecting an even balance across autistic and non-autistic dyads and in line with published recommendations for the chosen method of qualitative analysis [29].

**Table 1:** Demographics of the autistic and non-autistic dyads

	Autistic group		Non-autistic group	
	Individual with AN (n = 7)	Parent (n = 7)	Individual with AN (n = 7)	Parent (n = 7)
AN status	Current (n = 4), recovered (n = 3)		Current (n = 4), recovered (n = 3)	
Age in years (mean, SD)	24.14 (3.13)	54.86 (4.14)	24.58 (5.16)	55.83 (5.23)
Gender	Female (n = 6), male (n = 1)	Female (n = 7)	Female (n = 7)	Female (n = 5), male (n = 2)
Ethnicity	White (n = 7)	White (n = 7)	White (n = 7)	White (n = 7)
Level of education	Primary/secondary education (n = 4), Undergraduate degree (n = 1), postgraduate degree (n = 2)	Undergraduate degree (n = 3), vocational training (n = 1), doctorate or professional degree (n = 3)	Primary/secondary education (n = 1), undergraduate degree (n = 5), non-specified employment (n = 1)	Primary/secondary education (n = 2), postgraduate degree (n = 2), vocational training (n = 1), doctorate/professional degree (n = 2)
Level of employment	Student (n = 2), unemployed (n = 2), part-time employment (n = 1), full-time employment (n = 1)	Full-time employment (n = 3), self-employed (n = 1), full-time carer (n = 1), part-time employment (n = 1), retired (n = 1)	Student (n = 2), part-time employment (n = 2), full-time employment (n = 3)	Full-time employment (n = 3), self-employed (n = 1), part-time employment (n = 1), retired (n = 2)

### Procedure

Ethical approval was obtained from the University of Edinburgh (CLPS064). Participants were invited to contact the lead researcher if they were interested in taking part in the study. A participant information sheet was shared and contact details of the other member of the dyad were obtained. Once eligibility was confirmed and both members of the dyads had been given the opportunity to ask any questions, participants were asked to provide consent and complete a demographic survey using a secure online survey platform (Qualtrics). Dyads then participated in a semi-structured interview, lasting between 45 and 60 min on a secure online video platform (Zoom). Interview recordings were then transcribed and anonymised, assigning participants pseudonyms and removing all other names, locations, services or other possible identification sources.

### Interpretative phenomenological analysis

Semi-structured interviews were developed by the lead researcher and an Autistic researcher with lived/living experience of AN. Dyads were asked to provide a brief overview of how they received a diagnosis on anorexia and to identify any general factors or behaviours they thought affected their anorexia. Questions then focused specifically on the role of social and sensory factors. Prompts were also included for developmental perspectives from parents or caregivers, and specifically for the Autistic group with AN (please see Additional file 1 for interview schedule). Interview schedules guided by pre-determined questions, allowing participants flexibility to discuss their experiences, a key component of Interpretative Phenomenological Analysis (IPA; [30]). This approach was chosen as it acknowledges the complexity of experiences and allows for an in-depth analysis of the perceptions and experiences of individuals in smaller and purposive samples. IPA approaches are concerned more with how the individual experiences the phenomenon of interest, as opposed to producing an objective record of the phenomenon [31]. This approach is rooted within phenomenological and hermeneutic theoretical underpinnings, thereby seeking to understand and interpret the meaning of lived experiences [32].

IPA has typically been used in relatively homogenous samples of participants, looking to explore shared perspectives across individual participants regarding the phenomenon of interest. However, recent research has begun to use an idiographic IPA approach to exploring and synthesising multiple perspectives of the same phenomenon [33]. This allows researchers to move away from the potentially limiting nature of one-dimensional accounts and is useful for research questions that have a strong social and relational dimension [32]. Dyadic data has previously been analysed through an IPA lens with a focus on the pair as a pair [34, 35] and at a group level [36, 37]. Combining these approaches, an idiographic IPA approach was applied to each transcript individually, looking for emerging themes from the shared experiences within each dyad, before each dyad’s emerging themes eventually being integrated and analysed to generate superordinate themes across the group. This process was conducted twice; firstly, with the autistic adults and their parent’s group, and then again with the non-Autistic adults and their parents’ group.

The IPA process involves two stages of interpretation: firstly, participants attempt to make sense of their experience; and secondly, the researcher attempts to interpret and make sense of the participant making sense of their experiences. It is recognised that during this process, the researcher’s interpretation may be influenced by their own personal experiences and understandings. Drawing on this, the current study adopted a data triangulation approach [38], involving an Autistic researcher with lived/living experience of AN in the coding and development of the themes. This approach is rooted in calls for participatory research, both in autism [39] and in ED [40, 41] research, while also playing an important and novel role in the interpretation of experiences that is inherent to IPA. This data triangulation approach involved an Autistic researcher with lived/living experience of AN reviewing emerging themes with the dyads in each group, as well as emerging group themes. Several rounds of further analysis and discussions with the lead researcher were then conducted, before dyadic themes were finalised and merged into finalised superordinate group themes (please see Additional file 2 for a more detailed overview of the triangulation process).

### Researcher reflexivity

It is important here to include researchers’ reflexivity statements, reflecting on how expertise and experience may have affected the interpretation of the data. Two authors (EN, EM) were involved in the IPA process and thus it is important to consider possible influences on their personal interpretation of the data. Primary analysis was conducted by EN, a neurotypical PhD student who developed an interest in autism and disordered eating across her psychology undergraduate and postgraduate degrees. EN has also worked closely with the Autistic and ED community throughout her education and training and is keenly aware of both the complexity and the urgency associated with untangling autism and EDs. She is also mindful of the need for lived/living experiences in generating meaningful research. EM is an Autistic researcher with lived/living experience of AN and was involved in the coding and development of themes. EM has worked closely with families, clinicians, and Autistic and non-autistic individuals with lived/living experience of AN across various advocacy and policy projects, and has a special interest in understanding the overlap and differences between autism and AN.

## Results

IPA analysis revealed three superordinate themes across Autistic dyads: *The Social Self,* with sub-themes of *Different and ‘Defective’* and *Understood and (Un)United; Sensory Foundations;* and *Feeling the Unknown,* with sub-themes of *Emotion Sensitivity versus Expression* and *Interoceptive Sensitivity versus Expression.* Similarly, three superordinate themes were identified in non-autistic dyads: *The Social Self,* with sub-themes of *(In)adequacy and Acceptance* and *Over-empathy, Self-Apathy; Damaging Early Experiences,* with sub-themes of *Reinforcement of Body and Weight Ideals* and *Control-Seeking Behaviours;* and of *Distrust of the Bodily Self,* with sub-themes of *Interpreting Stomach Cues* and *Body Checking.*

**Fig. 1:**
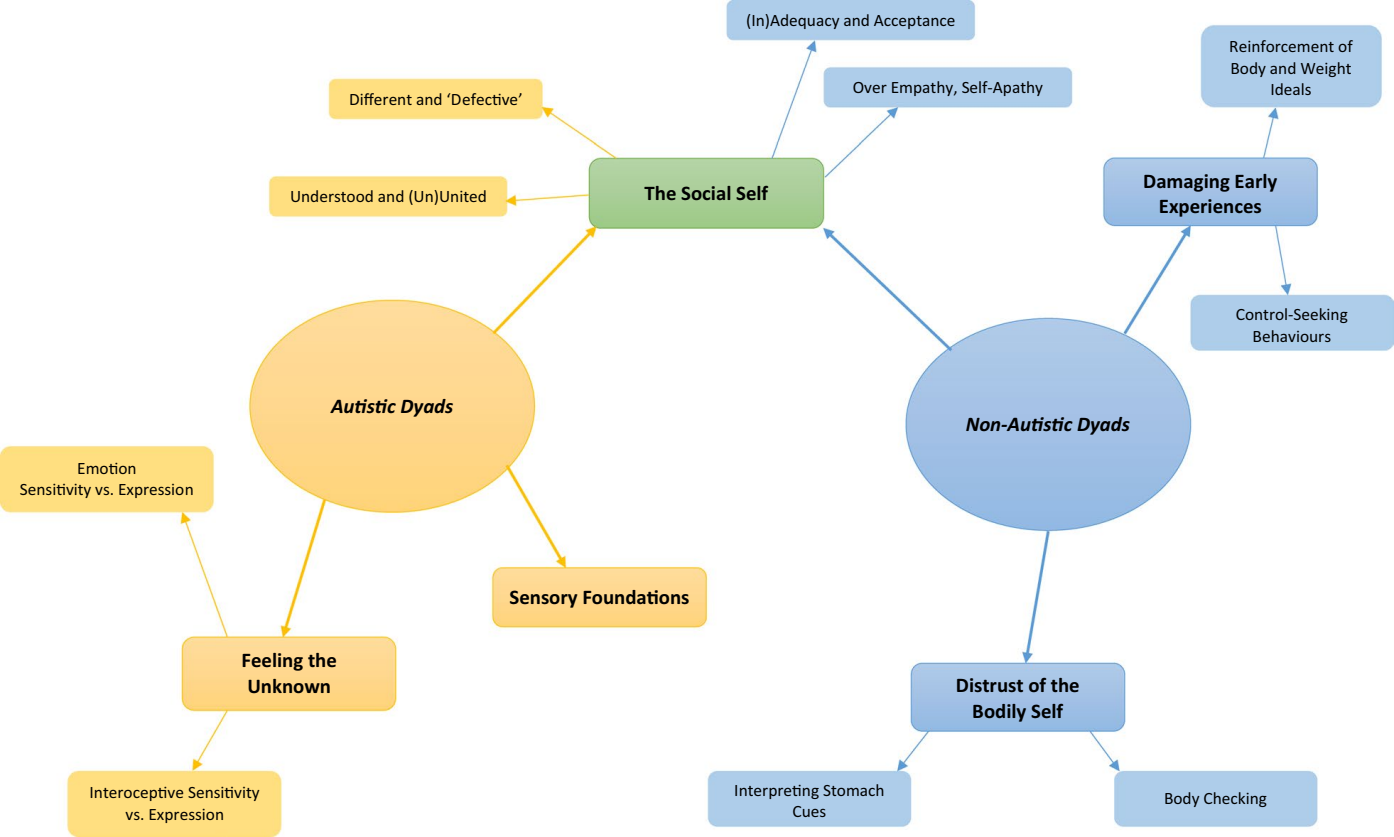
Visualisation of superordinate and sub-themes between groups

The following results section is a narrative synthesis of similarities and differences between autistic and non-autistic dyads across social and sensory factors. Pseudonyms are provided after each quote, with an A next to members of the Autistic dyads; for example, Holly(A). See Fig. 1 for a comparative summary of themes and sub-themes between autistic and non-autistic dyads.

### Social factors

A superordinate theme identified across both autistic and non-autistic dyads was *The Social Self.* This theme centred on a socially driven sense of self, with reflections on social experiences and connections playing a key role in how they defined themselves and their AN. However, within this shared superordinate theme were sub-themes that highlighted key group differences.

For Autistic dyads, this was defined by feelings of being different and therefore ‘defective’ (*Different and ‘Defective’).* This keen sense of difference to other people, mostly to their peers, led to feelings of social isolation and alienation. This was often perceived to be particularly notable in the immediate lead up to restrictive eating behaviours, suggesting that these particular social difficulties were a leading factor in the development of their AN. Often this was also accompanied by a certain degree of confusion, suggesting that it was not only the feeling of being different but the lack of understanding in what caused these differences that exerted an influence on their AN:

When I was in school, which was around the kind of the time I was diagnosed or just before, I can only realize now but how much I struggled with social interaction and kind of things like that. I’ve always felt really really different, and I didn’t really know why…just the way that I feel about myself, I think that’s a big part in feeling…um…I guess not feeling like I deserve or need to eat (Holly(A))

This combination gave rise to an internalized sense of ‘defectiveness’, of not only being different but of being to blame for this difference. This led in turn to disordered eating behaviours and thoughts. A lack of belonging and unworthiness was thought to play an integral role in the development and maintenance of anorexia by both members of the dyad:

I think the social aspect as much as anything, like Anita has never felt like she fitted in, she’s never felt like she’s had friends...I think a lot of it has stemmed from social interaction and not feeling like you’re not fitting in and not having what you think a friendship should be. [To Anita] do you agree? (Anita’s mum(A)) [Anita nodding]

The absence of social connections and feeling like they did not belong was perceived to be a lifelong experience; not something that was a consequence of the eating disorder but instead preceded and often influenced it. Furthermore, perceived disparity between individual’s expectations and the reality of their social interactions was a common experience across participants, feeding back into feelings of internalized inadequacy and inferiority. Subsequently, feelings of internalized unworthiness distorted their perceptions of other people’s emotions or intentions and led to a negative, self-biased interpretation of the world around them. This lens often led the individual to believe that they were cause of other people’s negative moods or behaviours:

If there is something wrong with somebody you’ll know right away...but it automatically causes you anxiety, because you’re first thought is what have I done wrong (Aaron’s mum(A))

There’s some distortion there like is occurring on my side. It’s not my ability to perceive those emotion but it’s just that distortion about myself and the way people feel about me that kind of interrupts the processing of that (Aaron(A))

Several participants reflected on the influence that receiving their autism diagnosis had on shedding light on their perceived social difficulties, although for many this was an ongoing process. Their autism diagnosis offered a lens through which to understand this entrenched sense of self-blame when it came to their feelings of social inadequacy:

I think when my diagnosis it was kind of a weight off my shoulders. Like okay with the socializing example, like I knew blaming myself…like I was doing something wrong and I was finding this more difficult than a normal person so clearly there is something wrong with me. And when I got the diagnosis it was like okay, I’ve not done anything wrong it’s just my brain works differently and I can now put things in place, try and like help myself in a way (Anita(A))

Also unique to Autistic dyads’ reflections on their social self was an emphasis on the protective role that feeling understood can have (*Understood and (Un)United).* For several dyads, this was discussed in the context of shared interests and ‘wiring’, and how meeting likeminded people help them to feel seen, heard and accepted. For some, this took a while for some participants to find, with one participant reflecting that this did not happen until she met others during treatment for her AN:

Actually, the first time I think since starting school I felt that I could relate to my peers was when there was a particular group of us in inpatient care… who I think were all wired the same way. (Madeline(A))

By which time you were how old? (Madeline’s mum(A))

“16” (Madeline(A))

16 before she could relate! (Madeline’s mum(A))

Once this was achieved, participants began to feel less misunderstood and isolated, and thus were able to begin renegotiating their social sense of self. On the flip side of this was the negative impact that an absence of shared wiring or connections can have, notably from a lack of understanding from non-Autistic individuals. This draws attention to a bi-directional pathway of communication between autistic and non-autistic individuals, whereby Autistic dyads felt more understood and thus more connected with other Autistic people, while non-Autistic people reinforced a sense of alienation and disconnectedness. A lack of autism understanding or awareness often came from healthcare professionals during ED treatment, where certain behaviours were misattributed to more neurotypical interpretations:

We have had so many non-autistic aware nurses and healthcare supporters and psychologists, even in the last period in hospital in the last year, saying you [Emma] are so rude. (Emma’s mum(A))

Yeah, I get that a lot. (Emma(A))

She did this once in a whole ward round….[so they said] what would you like to happen, and she said go home. Oh, that’s fantastic, we can see that Emma has got purpose she wants to get better, they start doing a plan and I’m like no logically Emma just wants to go home. She’s just answered the question!. (Emma’s mum(A))

This labelling of the participant as “rude” reflects a disparity between ED services’ understandings of disordered eating and autism-specific behaviours, ultimately leading to feelings of frustration within ED services. This only in turn served to negatively reinforce the individuals’ perceptions of being different, feeding back into this socially driven sense of self and ultimately into the manifestation of their AN.

Conversely, non-Autistic dyads reflected on a sense of social and self-inadequacy as being a key factor underlying the development and maintenance of their AN *((In)adequacy and Acceptance)*. This was predominantly driven by social comparisons, with participants frequently feeling like they were inferior or a failure:

But the summer before I became unwell…I was with a group of people that I didn’t know, and in that situation that’s when I started to hear what they’ve done, what they’re doing and that’s when I started to feel I’m not good enough. (Mia)

You had met other people who, you know, you felt were doing so much more than you were which again, I’m sure wasn’t true or wasn’t factual. (Mia’s mum)

Through this comparison, participants began to internalise a sense of inferiority, often distorted from reality. Social interactions and relationships therefore took on a significant role in defining the participants sense of self, and ultimately leading to the expression of their AN. Mirroring this, several other dyads discussed the restorative power that social connection and acceptance can have on the expression of their AN. This could come from feeling able to communicate within the family or with peers, as well as support services. Social acceptance was intertwined with self-worth, and influential in the expression of their AN:

And think just being liked more generally, feeling like people respond more positively to me as a person I think in some ways…makes me feel better about myself which makes it easier for me to be like the eating and exercise stuff feels less important because I’m like, well I’m a good person, like look, other people like me so this doesn’t really matter. (Zoe)

Disordered eating was commonly felt to be a means of social and emotional regulation; restrictive eating behaviours were perceived to have emerged in attempt to mitigate feelings of social inadequacy and to regulate the emotional impact this had on the individual. This emotional salience of social factors took on significance in making sense of their experiences of AN:

I was not allowed to restrict anymore but I just could not, did not know how to manage that impotence of doing everything I could think of and still feel alone or being abandoned, not appreciated. Most of my eating disorders is related to that (Rosie)

Another manifestation of a socially driven sense of self unique to non-Autistic dyads was reflections on being overly empathetic to the needs and emotions others, while simultaneously showing a lack of empathy towards the self (*Over-empathy, Self-apathy):*

And also, in terms of how she is with other people, you know, I think a couple of times your empathy... doesn’t get in the way, but you have to find that fine line between being empathetic and it having an impact on you. (Zoe’s mum)

Yeah, I remember finding it quite difficult living with someone else because I would be going like they’ll be finding this annoying, or they don’t want to hear this about me or I don’t want to bring up this thing that they’ve done because I don’t want to make them feel bad. And then I would be like oh great they’re making loads of noise at midnight again, but I don’t want to say anything because I don’t want to make them feel unsafe in our living environment (Zoe)

Often this was expressed as something that caused distress or interfered with the lives of individuals, suggestive of low self-worth and a lack of prioritising their own needs. There was a general sense that empathy was prevalent but often inaccurate, biased towards this hyper-focus on others and a hypo-focus on the self:

Hyper-able I would say [to identify emotions]… probably more so for other people. Like I’ll know if someone’s had enough talking to me, you know [laughing] I’ll know to wrap it up like. I’ll notice the signals, I’ll see the glance at the watch, I’ll see the eyes flittering to the left. I’ll pick up the anger signals, I’ll pick up if someone is upset very easily, yeah. (Louise)

I’m not sure…. That a part of being aware of other people’s emotions is possibly, ehm, going back to the whole notion of what people think of me...I’m not sure if there’s- there might be a little of that coming in there. (Louise’s dad)

Oh, there definitely is. (Louise)

A hyper-awareness and attuning to into other people’s emotions became integrated with a sense of self and social acceptance, before fundamentally being expressed through their AN:

And then when I was growing up, I gave up on being myself to be able to be loved, so I was doing whatever other people would do or what other people would expect from me, so as to get their attention and be loved. And it’s only now that I’m like trying, trying, not to care too much. To prioritize being myself as opposed to just accommodating what to what other people say. (Rosie).

Additionally unique to the non-Autistic group was a superordinate theme discussing the damaging influence of childhood experiences leading to the learning and expression of disordered eating behaviours (*Damaging Early Experiences).* For many dyads, this involved harmful exposure to weight, food and eating ideals (*Reinforcement of Weight and Eating Ideals).* Many of these discussions centred on the reinforcement of body or food shaming, coming from different social settings including schools, sports or at home:

There would have weighing scales in the changing room, there was weighing scales at our squad trainings and our weight was recorded. We used phrases like “I am a gymnast we do not eat sweets or chocolate” at every session at the beginning and end of the session. (Louise)

Through early exposure to these ideals, individuals learned to fear specific body shapes or weight gain from a young age. While the messages surround eating and body shape were explicit and thus consciously adopted, it is the implicit or unconscious learning of fear and shame that felt integral to the individuals experience of their subsequent AN. As a parent, this was felt to invoke guilt and a sense of having let down their child:

It was only years later that we heard “I’m a gymnast don’t eat sweets” We have been quite shocked that someone would do that, so it would make me very wary, something that I would say to my daughters who have children, to be very careful. (Louise’s dad)

A notable focus of several discussions was the influence of gender stereotypes. Weight and body shape were linked with societal expectations, and female participants felt the expectation to be thin. Many participants discussed this in the context of social factors, such as fitting in, being bullied at school, and learning to associate being accepted with being thin. These experiences were felt to be integral in the development of acceptance, as well as their experiences of AN:

For me it was like I felt that to be thin was to be loved so I started restricting a lot with food. (Rosie)

Another important dimension was the learning of emotions surrounding disordered eating behaviours. Several dyads discussed these experiences, focusing on the emotional associations with food and eating that they were exposed to early in their development:

Also, my mum…I think she has some form of an eating disorder. Not anorexia but sort of disordered eating behaviours, in terms of like being quite restrained in what she eats and sort of, ehm, don’t really know how to describe…like….a bit…I don’t know dad, how would you describe it? (Nina)

Anxious. Anxious about what she’s eating. (Nina’s dad)

I think just always the sort of sense that she was….one had to be cautious about what you ate, or one had to be careful not to eat too much and to only eat as much as you need, you know, that sort of thing. (Nina)

This extends beyond the learning of specific eating behaviours to subconscious internalisations of functions of an eating disorder, such as food restriction as an emotional coping skill. From an early age, exposure to a complex interplay between emotions and eating, gave rise to either a consciously or unconscious hypervigilance to food.

For other non-Autistic dyads, early social experiences were perceived to be damaging through the subconscious development of non-ED specific behaviours that were ultimately adopted as a means of seeking control within the context of their AN (*Control Seeking Behaviours).* These discussions focused on factors that were not specific to weight or food but were still perceived to be integral to their AN. This could take the form of bereavement, childhood trauma or mental illness in the family. Such experiences were felt to function as a leading driver behind the development of their AN, and the filter through which other disordered cognitions and behaviours developed from:

It was a coping mechanism that I knew. So I wasn’t grieving anything when I had anorexia but um, I was grieving a life that I didn’t have in a way, and the only coping mechanism was around food and it started off as a way of control and then it became wanting to be thin…the focus is always about my [alcoholic] dad and there was never anything about me. (Rebecca)

For another dyad, adverse academic pressures had a significant influence in how they made sense of AN:

You get pressures from the teachers being like you must revise, work hard, whatever, and I think from that being grilled into every day I kind of took it in a way that not everyone took it, and my brain must have just kind of took a different response…. If you don’t get the top grades employers aren’t going to want to see you, the better the grade the better your life will be. (Sophie)

So I haven’t heard some of the things Sophie has said tonight and for me, that teacher who said that comment, I’m really disgusted with and it kind of almost started with her school life? (Sophie’s mum)

These experiences often invoked feelings of distress and inadequacy in the individual, giving rise to control-seeking behaviours that then ultimately lead to the development of AN; for example, this participant went on to discuss how she then began to revise tirelessly, losing her sense of self and developing restrictive eating behaviours. There was a sense amongst dyads that the individual with AN was particularly vulnerable to these experiences, playing into perfectionistic and self-driven personalities, and that it was this combination that gave rise to disordered eating. Thus, for several dyads, these early experiences were felt to play an instrumental role in their experiences of AN, giving rise to behaviours that allowed them a sense of control in what was felt to be an uncontrolllable situation.

### Sensory factors

Notable differences were found in both the relative influence and the longevity of sensory processing differences between groups. Specifically, unique to Autistic dyads was the perception that sensory processing differences were a core factor, through which other factors (social, emotional, self) are influenced *(Sensory Foundations).* All seven dyads discussed lifelong sensitivities to a broad range of senses, including sound and touch, as well as to smell and taste, that were felt to be particularly salient to their disordered eating. It was not the nature of these sensitivities that were the focus of discussions however, but the response and impact that they had on the Autistic individual. Experiences of these sensory processing differences were deemed overwhelming by many, evoking a whole-body response:

Very uncomfortable. Not just like, I think with a lot of the sensory things it’s not just a case of a like or a dislike. I used to explain it as a full body response to it as opposed to oh I like that smell, I don’t like that smell. (Emma(A))

It’s like torture (Emma’s mum(A))

There was a keen sense across dyads that sensory sensitivities were highly emotive and inescapable experiences, and such factors were perceived to be the core lens through which all other aspects of their life were filtered. For example, sensory processing difficulties were reported to regulate social interactions:

It’s because social interaction is an extra thing to have like to try and process that I’m already putting a lot of my effort into that that other things will slip through easier. Like the lights will affect me more or people talking in the background will affect me more and that kind of stuff. (Anita(A))

This was thought to influence the expression of AN, in that that certain restrictive or rigid eating behaviours were attempts to regulate sensory stimulation. For example, one participant reflecting that her preferences for certain texture of foods lead to *“less mental noise” (Madeline(A)).* In time, these sensory- and regulatory-based eating preferences develop into disordered eating behaviours, creating a complex interplay between sensory processing and AN:

Her coping mechanism is the eating disorder. So, it actually goes the other way. Do you know what I mean? Not just the sensory being highlighted, the more impact there is on the eating disorder—I see it like a seesaw—I mean, more impact there is on the Autistic Emma coping with the world, the eating disorder struggles. (Emma’s mum(A))

Conversely, non-Autistic dyads did not discuss multi-sensory processing difficulties; instead, there was a general sense that any sensitivities, such as in taste or smell, were a consequence of the AN. For non-Autistic dyads, experiences of sensory processing differences were understood within the context of their AN and the resulting distrust of their body and of their self (*Distrust of the Bodily Self).* This was a key superordinate theme for non-Autistic groups and was notably discussed within the context of interoceptive cues, specifically that of hunger and fullness (*Interpreting Stomach Cues).* Dyads commonly reflected on the individuals ongoing attempts to make sense of internal bodily signals, particularly those related to hunger and fullness. Experiences of AN led participants to question hunger or fullness cues, evoking distrust not only of the signals themselves but their own ability to interpret them:

And it almost disrupts my knowing whether I feel hungry or if I’ve just got an uncomfortable tummy, so I could be hungry or I could not but I just don’t know because the overwhelming thing is just it feels uncomfortable. (Zoe)

That’s it. Because even quite recently you’ve said oh I don’t really feel hungry, you know, I don’t feel hungry. (Zoe’s mum)

This was evident across AN trajectories, with those in recovery noting that these signals remain salient and evocative, leaving them with a lingering sense of distrust:

Definitely hunger and fullness I find tricky, and I still don’t quite trust myself in terms of knowing whether a feeling is hunger or whether it’s just feeling a bit tired or sick... I don’t trust my feelings of fullness or hunger (Mia)

Discussions around these body signals reflected ongoing and collaborative sense making within the dyads, and their role in AN seemed less clear to the individual than social factors. However, there was a clear sense that this was a consequence of the AN and was more to do with a lack of trust in the self than with an underlying difficulty identifying the signal:

Before [AN], I always used to feel you know the hungry, the signs of when you're hungry or full up. During my anorexia I kind of lost those signs. Now I do get hunger signals, however, I can be hungry, like all the time, but I wouldn't recognize that. Well, I recognize that, but I chose to ignore it. (Sophie)

Another expression of this distrust in the sensory perceptions and experiences of the body was body checking behaviours (*Body Checking),* such as fixating on certain body parts, feeling for fat or bone or comparing pictures of the body to others. This was a personal, often internal experience to the individual and was typically expressed by the individual with AN as opposed to their parents. Body checking was often noted to cause maintenance behaviour, serving to reinforce disordered eating:

Sort of body checking [as maintenance]. So, looking in mirrors, feeling for bones over hips and over stomach, so certainly body checking would be one, a big one. (Mia)

For non-Autistic dyads, body checking was felt to be more of a safety or reassurance behaviour in light of an overarching distrust of the body, reflective of an attempt by the participant to regain trust with their physical body. Another example of this was comparing bodies and eating habits with peers as a means of indirectly seeking reassurance, both with regards to the self and in relation to their peers. The subtle nature of these body checking behaviours meant that they were harder to identify and thus harder to control:

I was in a friendship group with two other girls that had eating disorders….we almost bounced off each other. We were close, but we would compare each other without even noticing…it wasn't until one girl was so bad that she was hospitalized that we really realized. (Rebecca)

### The autistic lens of social and sensory experiences

Another superordinate theme that was uniquely identified in the Autistic group highlighted an experience of multi-dimensional confusion that spanned both socio-emotional and sensory factors. Specifically, this was an overarching sense of being able to sense or feeling sensations or emotions but feeling unable to identify or communicate them (*Feeling the Unknown).* While this was discussed separately both socio-emotional and sensory context, it is felt that this sense of confusion between cues and communication of cues was universal and integral to the Autistic experience.

Participants reflected on both their awareness of emotions and their sensitivity to emotions in social contexts *(Emotion Sensitivity vs. Expression)*. Many participants reputed having a heightened sensitivity to the emotions of others; in some cases, this was felt to be a *“a superpower” (Holly(A)).* However, while Autistic participants felt they were hyper-able to register emotions, difficulties were reported in identifying or naming what that emotion was. This often left the individual disorientated and distrustful of their interpretation of other people’s emotions too:

What I struggle with, and I struggle with it in my own life as much as anybody else’s, is actually knowing what to call this feeling and therefore what the solution to this feeling might be...I know people are down, I have seen down, but there is more than one route to down...And I can’t necessarily say this is down, I just know that this is not good... But I’m not very good at knowing how other, verbally, how other people are feeling. I just sort of feel like I absorb it like a sponge. And I sometimes feel in my own life, I often don’t know how to describe how I’m feeling, does that make sense? (Madeline(A))

Absorbing unidentifiable emotions was here felt to give rise to internal conflict, renewing feelings of confusion, distrust and frustration with the self. Similarly, some dyads discussed difficulties with the individual expressing their emotions to others, with subtle differences across participants around the perceived mechanisms underpinning this. Some dyads felt that this difficulty with expressing their emotions originates from this lack of self-identification, and that this became integrated with AN:

Um, expressing my emotions is very very difficult. Um, and that makes…that usually means that I kind of end up bottling them up. And I guess I know when I’m feeling bad, or I’m struggling or a lot, but kind of really pinpointing what I’m feeling, I just don’t really seem to be able to do that very well....trying to express how I feel or my emotions, that is very very hard for me. (Holly(A))

I think Holly does bottle things up and then things get really bad and she’ll have a bit of a meltdown, which is, you know, hard for her. And usually around the time of when she’s got to eat something (Holly’s mum(A))

For other dyads, a lack of emotional communication or expression was a form of emotional avoidance:

If someone asked how are you feeling, I would know exactly how I’m feeling, but I’m not going to tell them how I’m feeling. (Milly(A))

You have to ask the right questions (Milly’s mum(A))

I don’t want to communicate. I think it was because I knew it would be hard communicating how I was feeling, especially when things felt like a lot, I knew that if I answered truthfully then it would lead to more questions. And sometimes it felt easier to keep everything buried than to start working through how I was feeling (Milly’s mum(A))

Regardless of these subtle differences in underlying routes to emotion expression difficulties, participants universally reflected that they were integral to their experiences of AN, highlighting the enmeshed nature of emotions and disordered eating.

A similar phenomenon was also discussed with regards to sensory processing, particularly in the context of interoceptive cues *(Interoceptive Sensitivity vs. Identification)*. Participants reflected on their ability to register internal cues but expressed difficulty with labelling them. Some participants discussed this in the context of temperature or pain thresholds, while many dyads discussed the role of hunger and fullness cues:

So, I can tell you that I feel dizzy, that my stomach hurts, that I feel tired, that my mouth feels dry, but will not connect that to I feel hungry, or I feel thirsty. And I used, even when I was younger didn’t I, my eating would be on a routine so I knew that I had lunch so I would eat. It wasn’t like, oh I’m hungry (Emma(A))

Oh, can I have a biscuit (Emma’s mum(A))

And if I’ve eaten something, it was usually for the sensory seeking side of it? So, I’ve had time when I’ve wanted something crunchy so I’ve had something crunchy, it’s not like oh I’m hungry I’ll have a snack. I’ve never really done hungry. (Emma(A))

This was not felt to reflect a distrust in the signals themselves but an internalized distrust of their own interpretation of these signals. Thus, much like discussions around emotions, participants felt internal confusion and subsequent mistrust of the body and self:

I’m not very good at knowing when I’m hungry. It’s not necessarily that I don’t know when I’m hungry...it’s a bit hard sometimes to tell the difference? I know there’s a problem but I can’t tell you….I can’t tell you what the actual sensation is. Sometimes I can’t tell whether I’m really full or really hungry. (Madeline(A))

While dyads discussed pronounced and multi-faceted differences in interoception, it was notable that discussions around interpreting these internal bodily signals centred on hunger and fullness signals. The confusion that arose from these signals were both lifelong and salient to their experience of AN, suggesting that dyads felt this misinterpretation directly impacted their eating habits.

## Discussion

The current study explored the perceived role of social and sensory factors in AN across autistic and non-autistic adults and their parents. IPA analysis revealed three superordinate themes in each group, highlighting key similarities and differences across groups. Notably, while there were some similarities in social and sensory factors, such as socially driven sense of self and distrust in internal bodily sensations, there seems to be significant differences in experiences and manifestations of how social and sensory differences play a role in AN.

Both autistic and non-autistic dyads reflected on a socially driven sense of self that was perceived to play a significant role in the development and maintenance of their AN. This is line with existing evidence that suggests social and interpersonal difficulties are common in both autism [22] and AN [23], and that perceived social difficulties are integrally linked with the self-concept [42, 43]. However, differences emerged in possible underlying mechanisms for these difficulties. For the Autistic group, this was driven by a sense of internalized defectiveness, whereas for non-Autistic participants this was felt to reflect a sense of inadequacy through comparison to others. Previous literature has suggested differences, where social comparisons have been reported to play a significant role in AN pathology [23, 44], while difficulties with social interaction and feeling different have been proposed as an autism-specific mechanism in restrictive EDs [25]. Furthermore, a recent comparative study found evidence to suggest that some aspects of reciprocal social interaction may help discriminate between autistic and non-autistic individuals with AN [23], suggesting that autistic individuals have possibly unique difficulties in engaging with conversation or responding to social prompts. Current findings support these possible autism-specific differences in social interactions, suggesting that feeling of defectiveness is a significant factor causing these differences.

Both groups also discussed the protective role of belonging and feeling understood by others. This is supported in AN literature, where feeling connected to others and high-quality social relationships are associated with less severe ED pathology [45]. Social connectedness has received considerably less research attention in broader autism research, perhaps due to assumptions that Autistic people are socially withdrawn and lack social motivation [46]. Challenging this approach are Autistic lived perspectives, highlighting how important connections and friendships are to them [47, 48]. For participants in the current study, an important aspect of these connections was the concept of sharing or similar wiring. While not explicitly a social theory, Monotropism [49] is a theory accounting for intense interests and connections that could intuitively be applied to understanding social connections, particularly within the context of restrictive eating behaviours [25]. Future research should explore this, seeking to further understanding how these shared interests may influence autistic relationships and how this may play a role AN. Conversely, social interactions and connections were found to be negatively influenced a lack of understanding of Autistic interests or traits, leading to different communication styles. This is consistent with the Double Empathy theory [50], which suggest this mismatch in neurotypes (neurotypical vs neurodivergent) causes misunderstandings that result in difficult social interactions [51]. Our findings are also in line with recent qualitative research suggesting that connecting with other Autistic people has a positive impact on their wellbeing [52]. ED services should promote awareness of these possible differences, applying these understandings into the modification of treatment and ensuring that autistic individuals feel understood by support services.

Across both groups, emotion difficulties were integrally linked with their perceptions of their social relationships and felt to directly influence AN. Socio-emotional difficulties have consistently been implicated in AN [53] and difficulties in identifying, regulating and communicating emotions have been implicated in Autistic women with AN [18, 25]. By comparing the experiences of autistic and non-autistic individuals, the current study was able contextualise these similarities and work towards identifying different underlying mechanisms. While both groups reflected on difficulties expressing or communicating emotions, they differed in what they perceived to be the cause of these difficulties. Autistic dyads felt these difficulties were caused by an inability to identify the emotion, while non-Autistic participants believed these were caused by heightened consideration for others leading to a lack of consideration for expressing their own emotions. Future research could work towards further understanding how these different underlying perceptions and motivations may give rise to similar surface-level socio-emotional difficulties. Studies could adopt a mixed-methods approach, allowing comprehensive and complementary findings by generating clinically meaningful and measurable outcomes that are contextualised by lived/living experience perspectives [54].

Themes were identified across both groups focusing on the interpretation of internal body cues, particularly salient cues of hunger and fullness, in line with recent studies exploring interoception as a possible mechanism in disentangling autism and AN [55]. The unique qualitative approach of the current study allows us to add novel insights to autistic and non-autistic experiences of detecting or identifying hunger and fullness. Both groups reflected on confusion of these signals that ultimately led to a distrust of their body and of themselves. However, subtle differences were revealed in the perceived cause of these difficulties; for Autistic individuals, this was reported to be a lifelong and multi-dimensional difficulty, while for non-Autistic individuals this was felt to be a consequence of their AN. This has important implications for hunger and fullness cues as a target of ED interventions, suggesting both similar and different treatment targets. For example, both groups would benefit from approaches that foster trust and agency in the self, while for non-autistic participants particularly, ED clinicians should be aware of the personal and emotional significance of addressing these cues.

Domain-specific sensory processing differences, including taste and smell sensitivities, were reported to reflect a similar pattern across autistic and non-autistic groups. Non-Autistic dyads reported that these sensitivities emerging with the development of their AN, while Autistic individuals reflected on lifelong and multi-sensory sensitivities. Sensory processing differences were also felt to be an integral and overwhelming influence on how Autistic individuals see the world, impacting social and emotional functioning as well as the expression of their AN. This is consistent with high prevalence estimates of multi-sensory differences in autism [56] and evidence to support its influential role in socio-emotional difficulties [57] and restrictive eating behaviours [25, 58]. This highlights the importance of increasing awareness amongst ED clinicians regarding the heterogeneous nature of sensory processing differences in autism, and the need to modify of ED treatment to account for them in Autistic populations, as opposed to seeking to correct them.

There are several notable strengths of the current study. By using a qualitative approach, the study sought to generate understandings of social and sensory factors in AN that is rooted in lived/living experience, adhering to calls for the inclusion of lived/living experience within the design, implementation, interpretation and dissemination of ED research [41]. The study is also closely aligned with neurodiversity affirming frameworks and calls for more meaningful participatory research [39]. An Autistic researcher with lived/living experience of AN was involved in the design and development of the study, and the triangulation of neurotypical, neurodivergent and lived/living experiences perspectives in data analysis allowed for a robust interpretation of participant’s realities. Finally, while the study built on previous research that draws on multi-perspectives, the collection of these perspective in the current study through dyadic interviews in both groups, allowed for unique interactions in real time and allowed for a direct comparison between autistic and non-autistic groups.

Despite these strengths, the study is not without limitations. The dyadic approach could have created a recruitment bias, excluding participants who were either unable or unwilling to participant with a parent or caregiver. With regards to the sample, there was a female bias across both adult and parent participants. This female bias has been widely reported in the AN literature, as well as in research exploring the overlap between autism and AN [25]. Future studies should attempt to recruit more representative gender samples, including the under-researched non-binary and transgender autistic participant groups. Due to the comparative nature of the study between autistic and non-autistic individuals, the study did not account for important ED-specific factors, such as age of ED diagnosis, or compare across current and recovered groups. Both of these factors likely influence the experiences of the individual and should be included and explored in future studies with larger samples and more generalisable methodologies. Age of autism diagnosis was also not collected, which intuitively may play a similar role in the participants interpretation of their experiences and warrants future consideration.

The current study has several notable implications. The current study could be used as a foundation or guide for future studies including participatory approaches with lived/living experience researchers, particularly with regards to involving lived/living experience in the data analysis process. Furthermore, social and sensory factors identified in the current study should be explored in further research with larger and more heterogenous samples in future efforts to untangle the co-occurrence between autism and AN. Subject to such future research it is hoped that further elucidating the role of such factors will identify promising targets in ED interventions for both autistic and non-autistic individuals. Current findings suggest that feelings of defectiveness and difference should be addressed to alleviate both AN symptoms and broader socio-emotional functioning in Autistic people, and efforts should be made to accommodate for, as opposed to ‘fix’, sensory-based eating. Additionally, Autistic individuals may demonstrate different communication styles to non-Autistic individuals which need to be accepted and understood. This awareness should be incorporated towards improving the therapeutic alliance and delivery of interventions, ensuring that Autistic individuals feel understood by ED support services. This could also be translated into developing community-based support services, such as peer support, which could match Autistic individuals with Autistic mentors. Across both groups, interventions could target renegotiating a sense of trust with internal bodily signals, while being aware of potentially subtle differences in mechanisms; for example, the complex interplay with emotion should be accounted for in non-Autistic individuals, while Autistic individuals may need to rely on more external cues to correct an underlying difficulty in identifying such cues. More broadly, identifying, regulating and communicating emotions within social contexts may be a promising target for alleviating AN symptoms across both autistic and non-autistic groups.

## Conclusions

The current study explored and compared the role of social and sensory factors in AN across Autistic and non-Autistic dyads. Both groups were found to have a socially integrated sense of self, as well as reflecting on socio-emotional and sensory difficulties. Differences also emerged between groups, with Autistic dyads reflecting on feelings of social defectiveness, difficulties with identifying or naming emotions, and more enduring, invasive multi-sensory processing differences. This is different from the non-Autistic participants who were more vulnerable to social comparisons and influences, particularly during childhood, and felt a greater degree of social inadequacy. Future mixed-methods research using more representative samples should seek to further explore these factors, using lived/living experiences to contextualise clinically measurable ED outcomes. These studies could in turn help ED services and clinicians modify their practice or develop autism-informed interventions.

## Supplementary Information

**Additional file 1.** Semi-structured interview questions.

**Additional file 2.** Summary table of the idiographic IPA approach adopted for the study, including the role of the autistic researcher with lived experience of AN.

## Data Availability

The datasets used and/or analysed during the current study are available from the corresponding author on reasonable request.
