# Supplementary material for: “It’s not about wanting to be thin or look small, it’s about the way it feels”: an IPA analysis of social and sensory differences in autistic and non-autistic individuals with anorexia and their parents
Source: J Eat Disord. 2023 Jun 5;11:89. doi: 10.1186/s40337-023-00813-z (PMC10243074; doi:10.1186/s40337-023-00813-z)
Supplement: Supplementary file 1 — Additional file 1. Semi-structured interview questions. [file 40337_2023_813_MOESM1_ESM.docx]

**“*It’s not about wanting to be thin or look small, it’s about the way it feels”:* An IPA analysis of social and sensory differences in autistic and non-autistic individuals with anorexia and their parents**

**Semi-Structured Interview Questions**

Hello. I’m going to ask you some questions today about your experiences of having anorexia/having anorexia and being on the Autistic spectrum [*delete as appropriate*]. I’m particularly interested in the impact of any social or sensory differences with regard to any disordered eating. If it’s okay with you both, I will be recording this interview. This interview will be one of several that I’ll be combining to try and generate an over-arching idea of what factors could be affecting disordered eating behaviours or habits. Your names will be anonymised immediately after the interview, so your identities will be protected. We adhere to a strict data management and privacy plan, which you can find details about on your information sheet. Do you have any questions before we get started?

I just want to remind at this point that you have the right to withdraw at any point of the interview today, as well as up to 72-hours after the interview has finished. If this is the case, please let me or one of the other research supervisors know, contact details of which you will be provided with at the end of the interview. Are you happy to start?

I’ll address some questions to each of you directly, but please feel free to chip in if there is anything else you want to add to a particular question.

*Note to researchers: many of the questions are worded as if addressed to the autistic/non-autistic individual, due the focus being on their disordered eating and possible co-existing autism. Throughout the course of the interview, tailor questions for parent/caregiver perspectives e.g., did your child experience any sensory differences in early childhood that may have contributed to any disordered eating/social differences? Follow a general pattern of having one member of the dyad answer a question before addressing it to the other.*

**Topic 1: Experience of getting diagnosed with anorexia**

- Could you give me a brief overview of how you arrived at a diagnosis of anorexia?
  - FOR AUTISM AND AN: Which diagnosis came first?
  - Prompt parent/caregiver to contribute – maybe more information about early childhood, etc.
  - Prompt individual to provide a rough idea/definition of where they perceive to be at with their anorexia.

**Topic 2: General factors/behaviours**

- Can you think of any factors or behaviours that you think affects your anorexia? For example, following our discussion on your experiences of getting diagnosed with anorexia, what kind of factors do you think played a role in that?
- What about factors that are currently sustaining/sustained your anorexia? Are there any differences between how it started and how it is being maintained?
  - Development vs maintenance
- Is there anything that makes it worse?
- Is there anything that makes it better?
- FOR AUTISM AND AN: Are there different factors that affect your disordered eating and your autism? Are there any similar factors?

**Topic 3: Social**

- Do you think that there are any social factors that come into play for your disordered eating? Not so much interaction with others, more along the lines of ways in which you perceive other people and the world around you? This could be recognizing emotion, or being able to put yourselves in other people’s shoes?
  - Ideas for prompts: empathising abilities (if this comes up, ask about whether this changes between neurodiverse and neurotypical – probe double empathy); paying attention in social situations; emotional responses to social situation.
  - Specific prompt for parents/caregivers to provide a developmental perspective – any social differences in infancy/childhood?
- FOR AUTISM AND AN: Are there different social cognitive factors that affect your disordered eating and your autism? Are there any similar factors?

**Topic 4: Sensory**

- Do you have any differences in social processing?
  - Are you particularly sensitive to any colours/touch/tastes/sounds/smells? This can be either overly or underly sensitive - for example, are you particularly sensitive to any particular smells or tastes?
  - Do you actively seek out certain sensations – for example, do you have certain sounds or noises that you seek out? Certain smells?
  - Do you always know when you feel hungry or full? Hot or cold? Thirsty?
  - Parents/caregivers and a developmental perspective – any social differences in infancy/childhood?
- Do you think that any of these differences play an important role in your disordered eating?
- Do you think these differences play an important role in any of the social differences we discussed earlier?
