# Supplementary material for: “It’s not about wanting to be thin or look small, it’s about the way it feels”: an IPA analysis of social and sensory differences in autistic and non-autistic individuals with anorexia and their parents
Source: J Eat Disord. 2023 Jun 5;11:89. doi: 10.1186/s40337-023-00813-z (PMC10243074; doi:10.1186/s40337-023-00813-z)
Supplement: Supplementary file 2 — Additional file 2. Summary table of the idiographic IPA approach adopted for the study, including the role of the autistic researcher with lived experience of AN. [file 40337_2023_813_MOESM2_ESM.docx]

Summary table of the idiographic IPA approach adopted for the study, including the role of the autistic researcher with lived experience of AN.

| **IPA step** | **Details of each IPA step** | **Role of autistic researcher with lived experience of AN** |
| --- | --- | --- |
| Step 1: Looking for themes in the transcript | This step involves reading the transcript, re-reading the transcript, using the left-hand margin for note/points of interest and the right-hand margin for emerging themes |  |
| Step 2: Connecting themes in the transcript | This step involves listing emerging themes, clustering the themes into superordinate themes and producing a table of coherently ordered themes with supporting quotes | Reviews the emerging themes and quotes, providing feedback and/or alternative suggestions    Feedback is then integrated into subsequent revisions of dyadic themes |
| Step 3: Repeat process with all transcripts in group | This step involves repeating steps 1 and 2 for each dyadic transcripts in the first group of participants |  |
| Step 4: Create table of superordinate themes across group transcripts | This step involves a finalised table of superordinate themes that have emerged across the group from dyadic transcripts | Reviews the emerging themes and quotes, providing feedback and/or alternative suggestions    Feedback is then integrated into subsequent revisions of group themes |
| Step 5: Repeat previous steps for the second group of participants | This step involves repeating steps 1-4 for the second group of participants | Contributes to steps 2 and 4 outlined above for second group of participants |
| Step 6: Look for common and distinctive themes between groups of participants | This step refers to the write-up or presentation of the results, as opposed to the analysis; table of themes across groups are compared and similarities and differences are highlighted | Reviews and provides feedback until agreement is reached for final presentation of study results |
